# NOD2 Promotes Glioblastoma Progression Through Effects on Epithelial–Mesenchymal Transition and Cancer Stemness

**DOI:** 10.3390/biomedicines13082041

**Published:** 2025-08-21

**Authors:** Eshrat Jahan, Shubhash Chandra Chaudhary, S M Abdus Salam, Eun-Jung Ahn, Nah Ihm Kim, Tae-Young Jung, Jong-Hwan Park, Sung Sun Kim, Ji Young Lee, Kyung-Hwa Lee, Kyung-Sub Moon

**Affiliations:** 1 Departments of Neurosurgery, Chonnam National University Hwasun Hospital and Medical School, Hwasun 58128, Republic of Korea; eshratjahan2023@gmail.com (E.J.); dr.sm.a.salam@gmail.com (S.M.A.S.); happybear33@naver.com (E.-J.A.); jung-ty@jnu.ac.kr (T.-Y.J.); 2 Departments of Pathology, Chonnam National University Hwasun Hospital and Medical School, Hwasun 58128, Republic of Korea; schandrachaudhary@gmail.com (S.C.C.); mince1234@naver.com (N.I.K.); kimsspathology@jnu.ac.kr (S.S.K.); great0208@naver.com (J.Y.L.); 3 Laboratory Animal Medicine, College of Veterinary Medicine, Chonnam National University, Gwangju 61110, Republic of Korea; jonpark@chonnam.ac.kr; 4 BioMedical Sciences Graduate Program (BMSGP), Chonnam National University, Hwasun 58128, Republic of Korea

**Keywords:** cancer stem cells, disease progression, epithelial–mesenchymal transition, glioblastoma, NOD2 signaling adaptor protein

## Abstract

**Background**: Glioblastoma multiforme (GBM) represents one of the most aggressive and lethal primary brain malignancies, characterized by rapid proliferation, extensive invasiveness, and a dismal prognosis. Emerging evidence implicates nucleotide-binding oligomerization domain-containing protein 2 (*NOD2*), an intracellular pattern recognition receptor, as a potential driver of GBM progression. This study investigates NOD2’s role in promoting glioblastoma through its effects on the epithelial–mesenchymal transition (EMT) and cancer stem cell (CSC) markers. **Methods**: NOD2 expression levels and survival outcomes were assessed using TCGA data from GBM tumor samples (*n* = 153) and normal brain tissues (*n* = 5). NOD2 protein expression was validated in glioma cell lines using Western blot and immunofluorescence analyses. Functional studies employed siRNA-mediated *NOD2* knockdown to evaluate effects on cellular proliferation, migration, invasion, and colony formation, while correlations between NOD2 and EMT/CSC markers were assessed. **Results**: The analysis of TCGA data revealed a significantly elevated *NOD2* expression in GBM tumors compared to normal brain tissue, with a high *NOD2* expression correlating with a reduced disease-free survival in GBM patients. All tested glioma cell lines demonstrated robust NOD2 expression. Functional analyses demonstrated that NOD2 depletion substantially impaired cellular proliferation, migration, invasion, and the colony-forming capacity. Mechanistically, siRNA-mediated *NOD2* knockdown significantly decreased the expression of EMT (Snail, SLUG, Vimentin) and CSC markers (CD44, CD133) at both protein and mRNA levels. **Conclusions**: Our results indicate that NOD2 contributes to GBM progression by influencing EMT and CSC pathways. These findings suggest NOD2’s potential as a therapeutic target in glioblastoma, highlighting the need for further mechanistic studies and therapeutic exploration.

## 1. Introduction

Glioblastoma multiform (GBM) represents one of the most aggressive and lethal primary brain malignancies, characterized by rapid proliferation, extensive invasiveness, and a dismal prognosis. Despite therapeutic advances, the median survival rate for GBM patients remains less than one year [1]. Conventional treatments including chemotherapy and radiotherapy have shown limited improvement over the past two decades. While immunotherapy has revolutionized treatment approaches in various cancer types, combination strategies incorporating immunotherapy with radiotherapy or chemotherapy may offer promising avenues for GBM management [2].

Pathogen-associated molecular patterns (PAMPs) and damage-associated molecular patterns (DAMPs) significantly influence cancer progression by modulating immune responses and the tumor microenvironment [3,4]. These molecular patterns are recognized by pattern recognition receptors (PRRs), among which, NOD-Like Receptors (NLRs) function as cytoplasmic sensors of pathogenic threats and cellular stress [5]. Emerging evidence indicates that aberrant NLR signaling, driven by chronic inflammation, contributes to cancer progression [6]. Nucleotide-binding oligomerization domain-containing protein 1 and 2 (NOD1 and NOD2) play pivotal roles in immune system regulation [7]. NOD2, an intracellular PRR, mediates pathogen recognition and activates numerous biochemical processes within host immune cells [8]. Recent investigations have implicated NOD2 in the progression of colorectal, breast, lung, endometrial, and cervical cancers, correlating with enhanced tumor invasion, proliferation, and poor patient survival [9,10,11,12,13]. NOD2 orchestrates diverse cellular processes through interactions with microbial components and the subsequent activation of signaling cascades, including nuclear factor kappa B (NF-κb) and mitogen-activated protein kinases (MAPKs) [14].

The epithelial–mesenchymal transition (EMT) represents a fundamental biological process wherein epithelial cells acquire mesenchymal traits, resulting in enhanced migratory and invasive capacities [15]. This process, along with cancer stemness, constitutes critical mechanisms underlying malignant behavior across various cancer types. The EMT and cancer stemness are pivotal drivers of cancer metastasis and therapeutic resistance, consistently correlating with adverse clinical outcomes. Cancer stem cell (CSC) markers, including CD133 and CD44, characterize the CSC phenotype associated with tumor initiation, treatment resistance, and cancer recurrence [16].

Recent studies have demonstrated that the EMT represents a fundamental mechanism driving glioblastoma progression, with key transcription factors such as Twist, Snail, and Slug orchestrating this transition [17,18]. The glioblastoma microenvironment has been shown to promote EMT through glioma-associated microglia/macrophages and TGF-β signaling, with EMT induction linked to cancer stemness acquisition [17,19]. While NOD2’s role in cancer progression has been established across multiple tumor types, no previous study has specifically investigated the relationship between NOD2 expression and EMT processes in glioblastoma progression.

Given NOD2’s established role in cancer progression and the fundamental importance of the EMT and CSC in malignant behavior, we hypothesized that NOD2 may play a crucial role in GBM progression. Furthermore, we sought to investigate whether NOD2’s oncogenic effects in GBM might be mediated through its influence on EMT and CSC programs. Therefore, this study aims to comprehensively investigate NOD2’s functional role in GBM progression through a systematic analysis of its effects on proliferation, migration, and invasion and its potential influence on EMT and CSC markers, with the ultimate goal of establishing NOD2 as a potential therapeutic target for GBM treatment. Understanding the relationship between NOD2 and these key malignancy drivers could provide valuable insights into the glioblastoma pathogenesis and identify novel therapeutic opportunities.

## 2. Materials and Methods

### 2.1. TCGA (The Cancer Genome Atlas) Data Analysis

*NOD2* expression levels were analyzed across normal brain tissue and GBM specimens using the TCGAplot package (version 8.0.0) implemented in R statistical software. The dataset comprised 153 GBM tumor samples and 5 normal brain tissue controls from TCGA database. Survival analysis was performed using the Gene Expression Profiling Interactive Analysis 2 (GEPIA2) web-based platform (http://gepia2.cancer-pku.cn/, accessed on 22 May 2025). Patients were stratified into high and low *NOD2* expression groups, and both disease-free survival and overall survival outcomes were assessed using Kaplan–Meier analysis with log-rank statistical testing. Correlation analyses between *NOD2* expression and genes associated with EMT and CSC markers were conducted using the Tumor Immune Estimation Resource 2 (TIMER2) online database (http://timer.cistrome.org/, accessed on 22 May 2025). Pearson correlation coefficients were calculated to determine the strength and significance of these associations.

### 2.2. Cell Culture

GBM cell lines including the murine (GL261), kindly provided by Dr. Maciej S. Lesniak (Northwestern University, Chicago, IL, USA), and human cell lines (LN18, LN229, U251, U87 MG, T98 G), purchased from the American Type Culture Collection (ATCC; Manassas, VA, USA), were maintained in Dulbecco’s modified Eagle’s medium (DMEM), supplemented with 10% fetal bovine serum (FBS) and 1% penicillin–streptomycin. Cells were cultured in a humidified incubator at 37 °C with 5% CO_2_ atmosphere.

### 2.3. siRNA Transfection and Gene Knockdown

siRNA oligonucleotides targeting *NOD2* were synthesized by Bioneer (Daejeon, Republic of Korea), with sequence information detailed in Supplementary Table S1. For transfection, GBM cell lines (GL261, LN18, LN229, U251, U87 MG) were seeded at a density of 1.5 × 10^5^ cells per well in 6-well plates 24 h prior to transfection. Transfection complexes were prepared by incubating siRNA oligonucleotides with Lipofectamine RNAiMax reagent (Thermo Fisher Scientific, Waltham, MA, USA) for 5 min at room temperature according to the manufacturer’s protocol. The transfection complexes were then added to the cultured cells, and knockdown efficiency was assessed after 48–72 h of incubation. *NOD2* silencing was validated at both mRNA and protein levels using Western blot analysis and quantitative real-time PCR, respectively.

### 2.4. Western Blot Analysis

Total protein was extracted from glioma cell lines using radioimmunoprecipitation assay (RIPA) buffer (Biosolution, Suwon, Republic of Korea) containing 50 mM Tris-HCl (pH 7.5), 150 mM NaCl, 0.5% sodium deoxycholate, 2 mM EDTA, 1% Triton X−100, and 0.1% SDS. Protein concentrations were quantified using the TaKaRa BCA Protein Assay Kit (TaKaRa, Cat# T9300 A, Shiga, Japan), and 25–40 µg of protein was loaded per lane. Proteins were separated by 8–12% sodium dodecyl sulfate–polyacrylamide gel electrophoresis (SDS-PAGE) and then transferred onto polyvinylidene difluoride (PVDF) membranes (Millipore, Burlington, MA, USA). Membranes were blocked with 5% non-fat dry milk in Tris-buffered saline containing 0.1% Tween−20 to prevent non-specific binding.

Primary antibodies were applied overnight at 4 °C with gentle agitation (12 rpm) at the following dilutions: NOD2 (1:1000, Cat# ab36836, Abcam, Cambridge, UK), CD133 (1:1000, Cat# ab19898, Abcam), CD44 (1:1000, Cat# ab189524, Abcam), Snail (1:1000, Cat# 3879 S, Cell Signaling Technology, Danvers, MA, USA), Slug (1:1000, Cat# 9585 S, Cell Signaling Technology), Vimentin (1:1000, Cat# ab8987, Abcam), and β-Actin (1:5000, Cat# 3700 S, Cell Signaling Technology) as a loading control. Following primary antibody incubation and washing, membranes were incubated with horseradish peroxidase (HRP)-conjugated secondary antibodies for 1–2 h at room temperature. Secondary antibodies included anti-rabbit IgG (1:5000, Cat# 7074 S, Cell Signaling Technology) and anti-mouse IgG (1:5000, Cat# 7076 S, Cell Signaling Technology). Protein bands were visualized using enhanced chemiluminescence substrate (Immobilon Western, Millipore) and detected with the Amersham Imager 600 system (GE Healthcare Life Sciences, Boston, MA, USA).

### 2.5. RNA Extraction and Quantitative Real-Time PCR Analysis

Total RNA was extracted from cultured cells using TRIzol reagent (Invitrogen, Carlsbad, CA, USA). RNA concentration and purity were assessed using a NanoDrop ND spectrophotometer (Thermo Fisher Scientific). First-strand cDNA was synthesized from 1 μg of total RNA using the GoScript Reverse Transcription System (Promega, Madison, WI, USA) following standard conditions.

Quantitative real-time PCR was performed using VeriQuest SYBR Green qPCR Master Mix (Affymetrix Inc., Santa Clara, CA, USA) on a CFX96 Touch Real-Time PCR Detection System (Bio-Rad, Hercules, CA, USA). Thermal cycling parameters included initial enzyme activation at 95 °C for 10 min, followed by 40 cycles of denaturation at 95 °C for 15 s, annealing at 60 °C for 30 s, and extension at 72 °C for 30 s. Relative gene expression levels were calculated using the 2^−ΔΔCT^ method with GAPDH serving as the internal reference gene. All reactions were performed in triplicate, and primer sequences are listed in Supplementary Table S2.

### 2.6. Immunofluorescence Microscopy

Glioma cell lines including murine GL261 and human cell lines (LN18, LN229, U251, U87 MG, T98 G) were seeded at a density of 4 × 10^3^ cells per well in 8-well Lab-Tek II Chamber Slide system (Cat# 154534, Thermo Scientific) and cultured for 48 h at 37 °C.

Cells were washed with phosphate-buffered saline (PBS) and fixed with 4% paraformaldehyde for 20 min at room temperature. Following fixation, cells were permeabilized with 0.1% Triton X−100 (Sigma-Aldrich, Munich, Germany) and blocked with 2% bovine serum albumin (BSA) (BioShop Canada Inc., Burlington, ON, Canada) in Tris-buffered saline containing 0.1% Tween 20 (TBST) for 30 min at room temperature. Cells were incubated overnight at 4 °C with gentle agitation (12 rpm) using primary antibody against NOD2 (1:250 dilution, Cat# ab36836, Abcam). After washing five times with PBS, cells were incubated with Alexa 568-conjugated secondary antibody (1:500 dilution, Cat# A11077, Thermo Scientific) for 1 h at room temperature. Nuclear counterstaining was performed using 4’,6-diamidino−2-phenylindole (DAPI) at 1:1000 dilution (0.1 μg/mL) for 20 min at room temperature. Slides were mounted using aqueous mounting media (Cat# S3020, DAKO, Santa Clara, CA, USA), and coverslips were sealed with nail polish. Fluorescence imaging was performed using an EVOS FL system (Thermo Scientific) at 10 × and 20 × magnifications for analysis and documentation.

### 2.7. Mouse Xenograft Models and Immunohistochemistry

Formalin-fixed, paraffin-embedded (FFPE) tumor tissue blocks generated from a previous study were utilized for this analysis [20]. All animal procedures received approval from the Institutional Animal Care and Use Committee of Chonnam National University Medical School (CNU IACUC-H−2017–58, approved on 20 August 2017). Animal housing and experimental procedures were conducted in compliance with the Guidelines for the Care and Use of Laboratory Animals (DHEW publication, NIH 80–23). Briefly, these archived samples included syngeneic brain tumors created by orthotopic implantation of GL261 murine glioma cells into C57 BL/6 mice. Additionally, FFPE blocks from BALB/c nude mice bearing human GBM xenografts (U87 MG and U251 cell lines) were kindly provided by Dr. Chung-Kwon Kim (Sungkyunkwan University, Suwon, Republic of Korea).

Tissue sections were prepared from FFPE blocks at 3 μm thickness and processed for immunohistochemical analysis using a Bond-Max Autostainer system (Leica Microsystems, Buffalo Grove, IL, USA). Antigen retrieval was performed using ER1 solution (Leica Microsystems) for 15 min, followed by incubation with anti-NOD2 primary antibody (1:200 dilution, Cat# ab36836, Abcam) according to standard automated staining protocols. Histological images were captured using a Nikon Eclipse 80 i microscope equipped with a DS-Ri2 digital camera system and analyzed with NIS-Elements F 4.60.00 software (Nikon Corporation, Tokyo, Japan).

### 2.8. Cell Proliferation Assessment Using Ki−67 Immunofluorescence Staining

To evaluate proliferative activity following *NOD2* knockdown, human GBM cell lines (LN229, U251) and the murine GL261 cell line were analyzed using Ki−67 immunofluorescence staining. Both scrambled control and *siNOD2*-transfected cells were seeded at a density of 4 × 10^3^ cells per well in 8-well Lab-Tek II Chamber Slide systems (Cat# 154534, Thermo Scientific) and cultured for 48 h at 37 °C.

Cells were then processed for immunofuorescence staining using Ki−67 primary antibody (Cat# SC−23900, Santa Cruz biotechnology, Dallas, TX, USA) and Alexa Flour 488 goat anti-mouse IgG (Cat#A11001, Thermo Fisher Scientific) to identify proliferating cells, following the immunofluorescence protocol described above. Mean fluorescence intensity was quantified using ImageJ software (version 1.53k) to determine proliferation indices between control and NOD2-depleted groups.

### 2.9. Colony Formation Assay

To assess clonogenic capacity, scrambled control and *siNOD2*-transfected glioma cells were seeded in 6-well plates at densities of 100 and 200 cells per well and maintained in culture for 14 days. Culture medium was refreshed every 3 days. Following the incubation period, cells were fixed with 100% methanol and stained with 5% crystal violet solution. Visible colonies containing more than 50 cells were manually counted under light microscopy. Data represent the mean of three independent experiments performed in triplicate.

### 2.10. Cell Migration Assay

To evaluate migratory capacity, scrambled control and *siNOD2*-transfected glioma cells were seeded in 6-well plates at a density of 1.5 × 10^5^ cells per well, in DMEM supplemented with 10% fetal bovine serum (FBS) and cultured until reaching approximately 90% confluence. Prior to migration analysis, cells were serum-starved overnight to synchronize cell cycle and minimize proliferation-mediated gap closure. Wound healing assays were performed by creating uniform linear scratches across the cell monolayer using sterile 200 µL pipette tips. Detached cells and debris were removed by washing twice with PBS. Migration was monitored by capturing phase-contrast images of the scratch area at 0, 24, and 48 h post-wounding using consistent microscopic fields. Wound closure was quantified by measuring the remaining gap area relative to the initial scratch width. Data represent the mean of three independent experiments.

### 2.11. Cell Invasion Assay

Cellular invasive capacity was assessed using Transwell invasion chambers (24-well format, Corning Inc., Corning, NY, USA) equipped with 8 μm pore membrane inserts pre-coated with 1% gelatin matrix. Scrambled control and *siNOD2*-transfected glioma cells were seeded into the upper chamber at a density of 8 × 10^3^ cells per well in serum-reduced DMEM supplemented with 5% FBS. The lower chamber contained DMEM supplemented with 10% FBS, which served as a chemoattractant to drive directional cell migration.

Following 48 h of incubation at 37 °C with 5% CO_2_, cells that successfully invaded through the matrix and adhered to the lower membrane surface were fixed with methanol for 5 min stained using the Diff-Quik staining protocol. Invasive cell numbers were quantified by counting stained cells in multiple microscopic fields using ImageJ software. Data represent the mean of three independent experiments.

### 2.12. Statistical Analysis

Statistical analyses were conducted using GraphPad Prism software (Version 8.01, GraphPad Software Inc., San Diego, CA, USA). Data are presented as mean ± standard error of the mean (SEM). For comparisons between two groups, unpaired Student’s *t*-tests were performed. Multiple group comparisons were analyzed using one-way or two-way analysis of variance (ANOVA) followed by Tukey’s or Bonferroni’s post hoc tests, respectively. Statistical significance was defined as *p* < 0.05 (*, *p* < 0.05; **, *p* < 0.01; ***, *p* < 0.001).

## 3. Results

### 3.1. NOD2 Overexpression Correlates with Poor Clinical Outcomes and Enhanced EMT/CSC Signatures in GBM Patients

**Figure 1:**
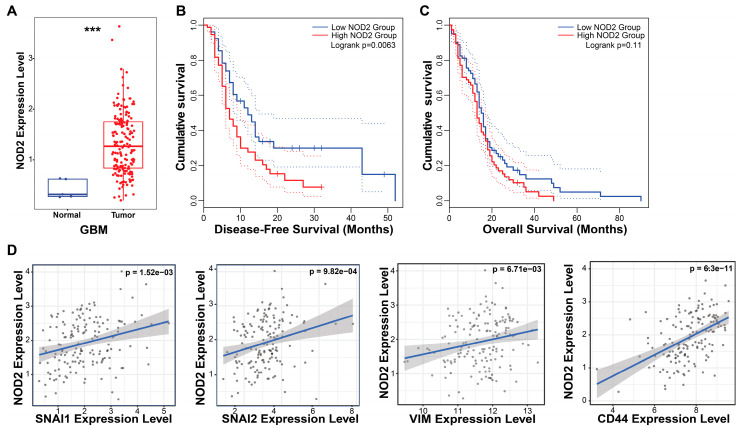
*NOD2* overexpression predicts poor prognosis and is associated with EMT and CSC signatures in GBM. (**A**) Bioinformatics analysis of TCGA data revealed significantly elevated *NOD2* expression levels in GBM tumor tissue (*n* = 153) compared to normal brain tissue (*n* = 5) (***, *p* < 0.001) (**B**) Kaplan–Meier survival analysis demonstrated that GBM patients with high *NOD2* expression exhibited significantly reduced disease-free survival (*p* = 0.007). (**C**) Kaplan–Meier curves showed a trend toward decreased overall survival in GBM patients with high *NOD2* expression, though this did not reach statistical significance (*p* = 0.11). (**D**) Correlation analysis revealed strong positive associations between *NOD2* expression and EMT markers (SNAI1, SNAI2, VIM) as well as the CSC marker CD44 (*p* < 0.001).

To investigate the clinical relevance of *NOD2* in GBM, we performed bioinformatic analyses using the TCGA database. Our analysis revealed a marked upregulation of *NOD2* transcript levels in GBM specimens (*n* = 153) relative to normal brain parenchyma (*n* = 5), indicating aberrant *NOD2* activation in malignant gliomas (Figure 1A).

The survival analysis demonstrated the prognostic significance of *NOD2* expression in GBM patients. The Kaplan–Meier analysis stratified by *NOD2* expression levels (measured in transcripts per million, TPM) revealed that patients harboring elevated *NOD2* expression experienced a significantly shortened disease-free survival (*p* = 0.007) (Figure 1B). Although the overall survival showed a similar trend toward worse outcomes in the high *NOD2* expression cohort, this association approached but did not achieve statistical significance (*p* = 0.11) (Figure 1C).

To elucidate the molecular mechanisms underlying *NOD2*’s association with a poor prognosis, we examined correlations between *NOD2* expression and key oncogenic pathways. Correlation analyses unveiled robust positive associations between *NOD2* levels and EMT regulators, including Snail (*SNAI1*), Slug (*SNAI2*), and Vimentin (*VIM*), as well as the CSC marker *CD44* (Figure 1D). These findings suggest that NOD2 may orchestrate aggressive tumor phenotypes through the coordinated activation of EMT and CSC programs.

### 3.2. GBM Cell Lines Exhibit NOD2 Overexpression and Respond Effectively to siRNA-Mediated Gene Silencing

**Figure 2:**
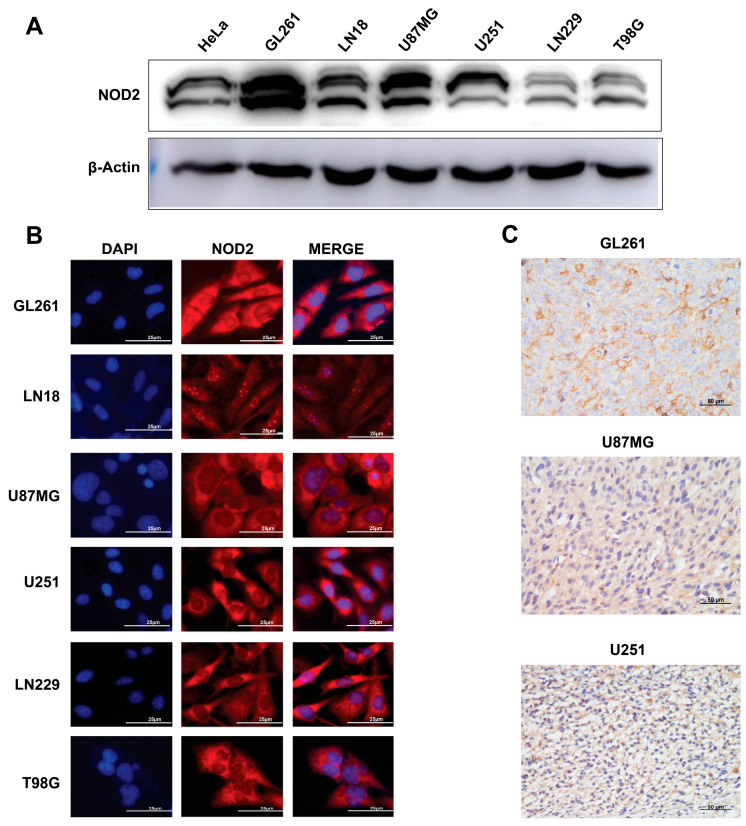
Baseline NOD2 expression in GBM cell lines and siRNA-mediated knockdown validation. (**A**) Western blot analysis revealed significantly elevated NOD2 expression levels in GBM cell lines (GL261, LN18, U87 MG, U251, LN229, and T98 G) compared to HeLa cells, which served as a positive control for NOD2 expression. (**B**) Immunofluorescence staining demonstrated NOD2 protein localization and expression patterns across the panel of GBM cell lines, including GL261, LN18, U87 MG, U251, LN229, and T98 G. (**C**) Immunohistochemical analysis of tumor tissue samples from mouse xenograft models confirmed NOD2 protein expression in GL261, U251, and U87 MG cell line-derived tumors.

To validate our TCGA findings in experimental models, we examined the NOD2 expression across human and murine GBM cell lines. The Western blot analysis demonstrated substantial NOD2 upregulation in all tested GBM cell lines (GL261, LN18, U87 MG, U251, LN229, and T98 G) when compared to HeLa cells (Figure 2A).

Immunofluorescence microscopy revealed a prominent cytoplasmic NOD2 protein distribution across all examined GBM cell lines (Figure 2B). This cytoplasmic localization is consistent with NOD2’s known function as an intracellular pattern recognition receptor. To further validate the NOD2 expression in vivo, we performed an immunohistochemical analysis on tumor specimens derived from mouse xenograft models. These analyses corroborated the NOD2 protein expression within tumor tissues generated from GL261, U251, and U87MG cell lines (Figure 2C).

**Figure 3:**
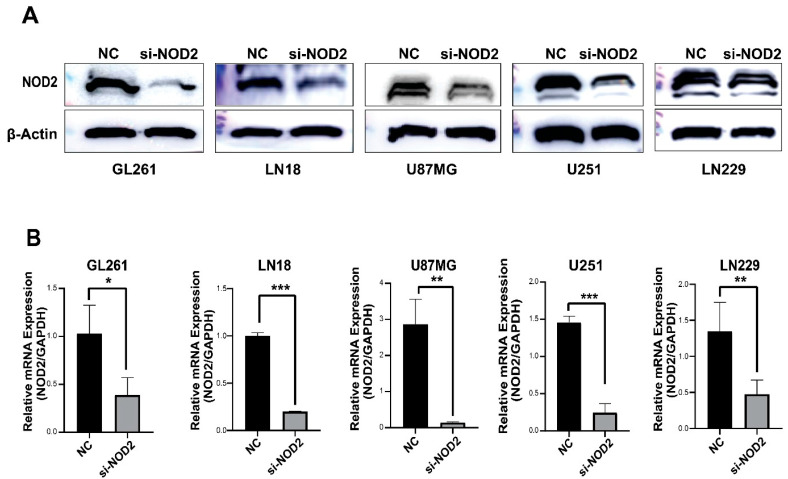
siRNA-mediated *NOD2* knockdown validation in GBM cell lines. (**A**) Western blot analysis validated the efficacy of siRNA-mediated *NOD2* knockdown, showing significantly reduced NOD2 protein levels in transfected GBM cell lines (GL261, LN18, U87 MG, U251, and LN229). (**B**) Quantitative real-time PCR (qRT-PCR) analysis confirmed significant reduction in *NOD2* mRNA expression following siRNA transfection, as depicted in the bar graphs (*, *p* < 0.05; **, *p* < 0.01; ***, *p* < 0.001).

The Western blot analysis confirmed a successful NOD2 protein reduction across all transfected GBM cell lines (GL261, LN18, U87 MG, U251, and LN229) (Figure 3A). The quantitative real-time PCR analysis further validated the knockdown efficiency at the transcriptional level (Figure 3B).

### 3.3. NOD2 Knockdown Leads to Coordinated Reduction in Malignant Phenotypes in GBM Cell Lines

**Figure 4:**
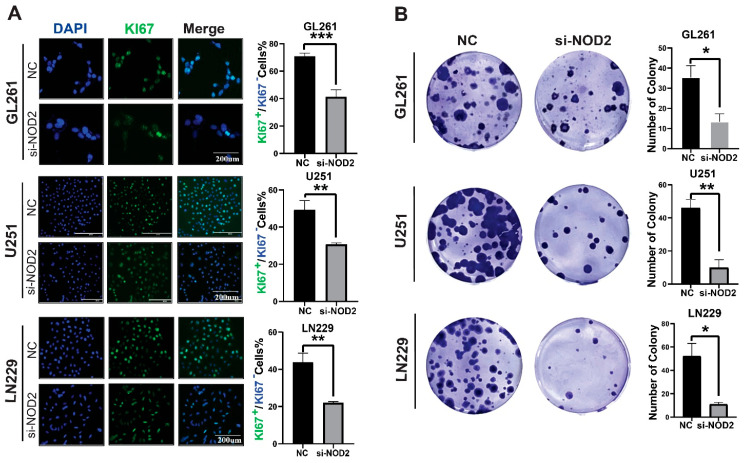
*NOD2* knockdown suppresses proliferation and colony formation in GBM cell lines. (**A**) Immunofluorescence analysis demonstrated that siRNA-mediated *NOD2* knockdown (*siNOD2*) significantly reduced Ki−67 proliferation indices in LN229, U251, and GL261 cell lines compared to scrambled control (NC) treatment. Mean fluorescence intensity was quantified using ImageJ software and represented in the corresponding bar graphs. (**B**) Colony formation assays revealed that *siNOD2* treatment substantially impaired the clonogenic capacity of LN229, U251, and GL261 cell lines relative to scrambled controls (NCs), as quantified and displayed in the bar graphs (*, *p* < 0.05; **, *p* < 0.01; ***, *p* < 0.001).

To elucidate the functional role of NOD2 in the glioblastoma pathogenesis, we conducted phenotypic analyses following gene silencing in multiple GBM cell lines. Ki−67 immunofluorescence staining revealed that the NOD2 depletion significantly attenuated the proliferative activity across LN229, U251, and GL261 cell lines (Figure 4A). Clonogenic assays further demonstrated that the NOD2 knockdown compromised the colony-forming capacity of all tested cell lines (Figure 4B).

**Figure 5:**
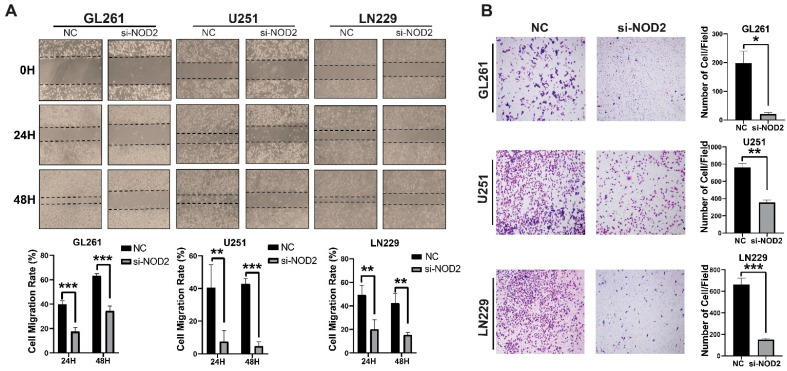
NOD2 knockdown suppresses invasion and migration capabilities in GBM cell lines. (**A**) Wound healing migration assays demonstrated that siRNA-mediated *NOD2* knockdown (*si-NOD2*) significantly impaired the migratory capacity of LN229, U251, and GL261 cell lines compared to scrambled control (NC) treatment, as quantified and represented in the corresponding bar graphs. (**B**) Transwell invasion assays revealed that siRNA-mediated *NOD2* knockdown (*si-NOD2*) substantially reduced the invasive potential of LN229, U251, and GL261 cell lines relative to scrambled controls (NCs) (original magnification, ×400), as quantified and displayed in the corresponding bar graphs (*, *p* < 0.05; **, *p* < 0.01; ***, *p* < 0.001).

Beyond proliferative effects, NOD2 silencing profoundly impacted the cellular motility and invasive behavior. Wound healing migration assays demonstrated a significant impairment of the migratory capacity in NOD2-depleted cells across all three cell lines tested (Figure 5A). Transwell invasion assays revealed that the NOD2 knockdown substantially diminished the invasive potential of LN229, U251, and GL261 cells (Figure 5B).

**Figure 6:**
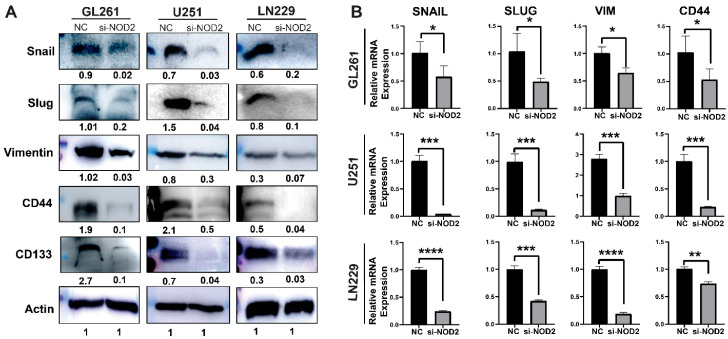
*NOD2* knockdown-mediated suppression of aggressive behavior associates with reduced EMT and CSC features. (**A**) Western blot analysis demonstrated that siRNA-mediated *NOD2* knockdown (*siNOD2*) significantly reduced protein expression levels of EMT markers (Snail, Slug, Vimentin) and CSC markers (CD44 and CD133) compared to scrambled control (NC) treatment. (**B**) Quantitative real-time PCR (qRT-PCR) analysis corroborated these findings, confirming decreased mRNA expression of EMT markers (Snail, Slug, Vimentin) and the stemness marker CD44 following NOD2 knockdown relative to scrambled controls (NCs) (*, *p* < 0.05; **, *p* < 0.01; ***, *p* < 0.001; ****, *p* < 0.0001).

To investigate the molecular mechanisms underlying these phenotypic changes, we examined the expression of key regulatory pathways associated with cancer aggressiveness. The Western blot analysis revealed that NOD2 knockdown significantly downregulated protein levels of critical EMT-related factors (Snail, Slug, and Vimentin), alongside the reduced expression of CSC markers CD44 and CD133 (Figure 6A). The quantitative real-time PCR analysis validated these protein-level changes, confirming the transcriptional suppression of EMT regulators (Snail, Slug, Vimentin) and the stemness marker CD44 following the *NOD2* depletion (Figure 6B).

Collectively, these findings suggest that NOD2 contributes to malignant phenotypes of GBM, including the upregulation of the proliferation, invasion, and migration, through EMT and CSC pathways.

## 4. Discussion

In this study, we comprehensively investigated NOD2’s role in GBM progression through the bioinformatics analysis of clinical datasets and functional characterization in glioma cell lines. Our findings demonstrate that NOD2 is significantly overexpressed in GBM tumors compared to normal brain tissue and correlates with poor patient survival outcomes. Functional studies revealed that NOD2 depletion substantially impairs key malignant behaviors, including proliferation, migration, invasion, and the clonogenic capacity. Mechanistically, we found that NOD2 influences the expression of EMT and CSC markers, suggesting its involvement in critical pathways underlying GBM aggressiveness.

NOD2, a cytoplasmic pattern recognition receptor primarily known for bacterial pathogen recognition and immune response activation [5], has recently emerged as an important player in cancer biology. While the brain’s immune-privileged status limits the PAMP exposure, alternative endogenous ligands may activate NOD2 signaling in the tumor microenvironment. Our TCGA analysis and immunohistochemical validation demonstrate a significant NOD2 upregulation in GBM specimens, suggesting that NOD2 contributes to the GBM pathogenesis beyond its conventional immune functions. This finding aligns with recent reports indicating that an aberrant NOD1/2 and NLRC4 expression correlates with poor survival in GBM patients [21,22].

The EMT represents a fundamental process driving cancer progression, wherein epithelial cells acquire mesenchymal traits that enhance invasiveness and therapeutic resistance [23]. Our functional analyses demonstrated that the NOD2 knockdown significantly reduced the expression of key EMT regulators, including the transcription factors Snail and Slug, as well as the mesenchymal marker Vimentin. This downregulation of EMT markers corresponded with impaired migratory and invasive capabilities in NOD2-depleted cells, suggesting that NOD2 promotes the mesenchymal phenotype that facilitates GBM cell invasion and therapeutic resistance.

CSCs constitute a critical subpopulation within tumors, possessing a self-renewal capacity and driving tumor initiation, progression, and recurrence [24,25,26]. Our experimental results indicate that NOD2 influences cancer stemness properties in GBM cells, as evidenced by the reduced expression of CSC markers CD44 and CD133 following the NOD2 knockdown. This reduction in stemness markers correlated with a diminished clonogenic capacity, suggesting that NOD2 contributes to maintaining the stem-like properties that confer tumorigenic potential and treatment resistance in GBM.

The mechanistic pathways through which NOD2 promotes the EMT and CSC in GBM involve various signaling cascades. While proper NOD2 signaling maintains immune cell homeostasis under physiological conditions [8], its aberrant activation in the tumor microenvironment may contribute to malignant progression through the altered cytokine, chemokine, and growth factor production that influences immune cell recruitment and function. Previous studies have identified the NOD1/2-NF-κb/ERK and IL−8 signaling axis as a potential driver of cancer progression in cutaneous squamous cell carcinoma [12], suggesting similar mechanisms may operate in GBM. Interestingly, NOD2’s role in cancer appears context-dependent, as evidenced by reports showing that NOD2 can inhibit tumorigenesis and enhance therapeutic efficacy through the AMPK pathway in hepatocellular cancer [7]. However, across multiple cancer types, including gastric, lung, and breast cancers, *NOD2* polymorphisms and altered expression have been associated with an increased cancer risk and poor patient survival [27,28,29]. These findings suggest that NOD2’s oncogenic or tumor-suppressive functions may be tissue-specific and dependent on the underlying molecular context.

Given these insights, targeting NOD2 represents a potentially novel therapeutic strategy for GBM treatment. The pharmacological inhibition of NOD2 or its downstream signaling pathways might effectively reduce the EMT activation, impair CSC properties, and consequently limit GBM invasion and proliferation. Further investigation of NOD2-targeted therapeutic approaches may yield promising treatment options for GBM patients.

## 5. Conclusions

Our findings demonstrate that NOD2 plays an important role in GBM progression through its effects on EMT and CSC markers. NOD2 overexpression correlates with poor patient outcomes and promotes key malignant behaviors including proliferation, migration, invasion, and the clonogenic capacity in GBM cells. Understanding the intricate mechanisms by which NOD2 influences these processes provides valuable insights into the GBM pathogenesis and highlights potential therapeutic opportunities. Continued research is essential to fully elucidate NOD2’s mechanistic role and to develop targeted therapeutic strategies that could improve clinical outcomes for GBM patients.

## Data Availability

The datasets used and analyzed during the current study are available from the corresponding author on reasonable request.

## Supplementary Materials

The following supporting information can be downloaded at https://www.mdpi.com/article/10.3390/biomedicines13082041/s1: Supplementary Table S1: siRNA Oligonucleotides for Mouse and Human *NOD2*. Supplementary Table S2: Primer Sequences for Mouse and Human Samples.

## Abbreviations

The following abbreviations are used in this manuscript:

NOD2	Nucleotide-binding oligomerization domain 2
CSC	Cancer stem cell
EMT	Epithelial–mesenchymal transition
TCGA	The Cancer Genome Atlas
GBM	Glioblastoma multiform
PAMPs	Pathogen-associated molecular patterns
DAMPs	Damage-associated molecular patterns
PRR	Pattern recognition receptor
NLR	NOD-like receptor
NF-ΚB	Nuclear factor kappa B
MAPKs	Mitogen-activated protein kinases
TPM	Transcripts per million
