# Supplementary material for: NOD2 Promotes Glioblastoma Progression Through Effects on Epithelial–Mesenchymal Transition and Cancer Stemness
Source: Biomedicines. 2025 Aug 21;13(8):2041. doi: 10.3390/biomedicines13082041 (PMC12383808; doi:10.3390/biomedicines13082041)
Supplement: Supplementary file 1 [file biomedicines-13-02041-s001.zip › SupplementryTableS1.pdf]

Supplementary Table S1. *si*RNA Oligonucleotides for Mouse and Human NOD2

Target *si*RNA ID	Cat#	Sense Strand (5' → 3')	Antisense Strand (5' → 3')
Mouse NOD2 *si*-1	257632-1	GAC GCU CUU CAA CCU U=tt(2-AS)	AAG GUU GAA GAG CAG AGU C 2-AA
Mouse NOD2 *si*-2	257632-2	CUG ACA ACA GAC UUC UGA A tt	UUC AGA AGU CUG UUG UCA G 1-AA
Mouse NOD2 *si*-3	257632-3	AGU UCC UGA AAC UUA CCU U tt	AAG GUA AUG UGC AGG AAC U 3-AA
Human NOD2 *si*-1	64127-1	CUG ACA GAC UUC UGA A tt	UUC AGA GAC UUG UUG UCA G 1-AA
Human NOD2 *si*-2	64127-2	CAG AGA GUA GCU CUA UUC A tt	UGA AUA CUC GAG CAU ACU UCU G 1-AA
Human NOD2 *si*-3	64127-3	CUC CAU UGC UAA GCU CCU U tt	AAG GAG GCU AGC CAU GGA G 3-AA
