# Supplementary material for: NOD2 Promotes Glioblastoma Progression Through Effects on Epithelial–Mesenchymal Transition and Cancer Stemness
Source: Biomedicines. 2025 Aug 21;13(8):2041. doi: 10.3390/biomedicines13082041 (PMC12383808; doi:10.3390/biomedicines13082041)
Supplement: Supplementary file 1 [file biomedicines-13-02041-s001.zip › SupplementryTableS2.pdf]

Supplementary Table S2. Primer Sequences for Mouse and Human Samples

Gene	Species	Forward Primer (5' - 3')	Reverse Primer (5' - 3')
NOD2	Mouse	GAGGGCTTTGAGAGCATCTTA	CTCTGAGACGACGTGAAGATT
CD133	Mouse	CTCATGCTTGAGAGATCAGGC	CGTTGAGGAAGATGTGCACC
CD44	Mouse	CTGTAGTGAAACACAACACC	GAAAGGCATCTTATGGATGTGC
SNAIL	Mouse	GCCGGAAGCCCCAACTATAGCGA	TTCAGAGCGCCAGGCTGAGGTACT
SLUG	Mouse	CGCGAATTCCCGCCCGCAGCCACC	ACTCTCGAGCTAGTGTCAATGGGCGAC
VIMENTIN	Mouse	CTGAGGCTGCCAACCGGAACAA	CCTCGCCTTCCAGCAGCTTCC
GAPDH	Mouse	TCACCACCATGGAGAAGGC	GCTAAGCAGTTGGTGGTGAC
NOD2	Human	CTCAGCTTCCCAAGGTCTGG	AGGTAGAACGCGGCAAAGAA
CD44	Human	TGAATATAACCTGCCGCTTTG	TCCGTCCGAGAGATGCTGTAG
SNAIL	Human	TCGGAAGCCTAACTACAGCGA	AGATGAGCATTGGCGCGAG
SLUG	Human	CGAACTGGACACACATACAGTG	CTGAGGATCTCTGGTTGTGG1
VIMENTIN	Human	GACGCCATCAACACCGAGTT	CTTTGTCGTTGGTTAGCTGGT
GAPDH	Human	GAAGGTGAAGGTCGGAGTCA	TTGAGGTCAATGAAGGGGTC
